# An Example of an Improvable Rao–Blackwell Improvement, Inefficient Maximum Likelihood Estimator, and Unbiased Generalized Bayes Estimator

**DOI:** 10.1080/00031305.2015.1100683

**Published:** 2016-03-31

**Authors:** Tal Galili, Isaac Meilijson

**Keywords:** Improper prior, Minimal sufficiency, Uniform distribution, Uniformly minimum-variance unbiased estimator.

## Abstract

The Rao–Blackwell theorem offers a procedure for converting a crude unbiased estimator of a parameter θ into a “better” one, in fact unique and optimal if the improvement is based on a minimal sufficient statistic that is complete. In contrast, behind every minimal sufficient statistic that is not complete, there is an improvable Rao–Blackwell improvement. This is illustrated via a simple example based on the uniform distribution, in which a rather natural Rao–Blackwell improvement is uniformly improvable. Furthermore, in this example the maximum likelihood estimator is inefficient, and an unbiased generalized Bayes estimator performs exceptionally well. Counterexamples of this sort can be useful didactic tools for explaining the true nature of a methodology and possible consequences when some of the assumptions are violated.

[Received December 2014. Revised September 2015.]

## 1.INTRODUCTION

Statistical theory courses usually start with basic notions for describing how much information on some unknown parameter θ can be obtained from a set of data *Y*. These are likelihood, sufficiency, minimal sufficiency, completeness, the Rao–Blackwell (Rao 1945; Blackwell 1947), and Lehmann–Scheffé theorems (Lehmann and Scheffé 1950, 1955). Familiarity with these notions is assumed.

The Rao–Blackwell theorem (RBT) offers a procedure (coined “Rao-Blackwellization” seemingly by Berkson 1955) for improving a crude unbiased estimator *g*(*Y*) of the parameter θ into a better one (in mean-squared-error or any other convex loss function), by taking the conditional expectation of *g*(*Y*) given some sufficient statistic *T* = *T*(*Y*), that is, ${{\hat{\theta }}_{\text{RB}}} = {E_\theta }\left[ {g\left(Y \right)|T} \right]$ (this is a statistic because *T* is sufficient). The Lehmann–Scheffé theorem and RBT taken together state that the (unique) unbiased estimator based on a *complete* minimal sufficient statistic *T* achieves uniformly smaller expected loss under any convex loss function (the common term UMVUE, uniformly minimum-variance unbiased estimator, stresses only squared loss). Furthermore, if a parameter can at all be unbiasedly estimated, then it can also be unbiasedly estimated by a function of *T*, and the Rao–Blackwell improvement of the former automatically leads to the latter. If an unbiased estimator of a parameter is *not* a function of the sufficient statistic *T*, the Rao–Blackwell improvement based on *T* is a strict improvement over the original unbiased estimator.

Classical examples for these ideas are often based on unconstrained exponential families of distributions in which a complete sufficient statistic always exists, and the Rao–Blackwell improvement yields optimal unbiased results (see Abramovich and Ritov 2013). However, applying Rao–Blackwell improvements with *noncomplete* minimal sufficient statistic *T* will always yield some estimator that fails to have minimal possible variance, at least somewhere in the parameter space: Any non-degenerate function of *T* with mean identically zero (the existence of such is the definition of noncompleteness) is an unbiased estimator of the zero function that is left unchanged by Rao-Blackwellization because it is already a function of *T*, but is dominated (with strict variance inequality somewhere) by the zero statistic.

The didactically motivated family of examples to be introduced does not depend on delicate measure-theoretical pathologies of noncountably generated families of distributions. It deals instead with commonplace positive uniformly distributed random variables parameterized by their mean, and yields a *uniformly* dominated Rao–Blackwell improvement. Consider the uniform distributions *U*((1 − *k*)θ, (1 + *k*)θ) with unknown mean θ > 0 and known design parameter *k* ∈ (0, 1), henceforth the *scale-uniform* family of distributions. This family does not satisfy the usual differentiability assumptions leading to Fisher Information, the Crámer-Rao bound and efficiency of maximum likelihood estimators (MLEs). For this family, MLE is inefficient.

While proper Bayes estimators cannot be unbiased (Bickel and Blackwell 1967), unbiased examples have been built under improper priors, such as the sample mean in the normal case. An explicit Bayes estimator built under a specific improper prior will be shown to be unbiased, simultaneously for all sample sizes and all *k*.

## 2.AN IMPROVABLE RAO–BLACKWELL IMPROVEMENT

Let *X*_1_, *X*_2_, …, *X_n_* be a random sample from a scale-uniform distribution *X* ∼ *U*((1 − *k*)θ, (1 + *k*)θ), with unknown mean *E*[*X*] = θ and known design parameter *k* ∈ (0, 1). In the search for “best” possible unbiased estimators for θ, it is natural to consider *X*_1_ as an initial (crude) unbiased estimator for θ and then try to improve it. Since *X*_1_ is not a function of *T* = (*X*_(1)_, *X*_(*n*)_), the minimal sufficient statistic for θ (where *X*_(1)_ = min (*X_i_*) and *X*_(*n*)_ = max (*X_i_*)), it may be improved using the Rao–Blackwell theorem as follows:

(1) θ^RB=Eθ[X1|X1,Xn]=X(1)+X(n)2\begin{eqnarray*} {\hat{\theta }_{\text{RB}}} = E_\theta [ {{X_1}|{X_{\left(1 \right)}},{X_{\left(n \right)}}} ] = \frac{{{X_{(1)}} + {X_{(n)}}}}{2} \end{eqnarray*}

since *X*_1_|*T* ∼ *U*(*X*_(1)_, *X*_(*n*)_). Although this estimator has lower variance than *X*_1_ (and $\overline{X}_n$), it is not even best among the linear unbiased estimators for θ. For this family of uniform distributions, there exists an unbiased estimator ${\hat{\theta }_{\text{LV}}}$ for θ with uniformly lower variance than the Rao–Blackwell improvement of *X*_1_. Both ${\hat{\theta }_{\text{LV}}}$ and ${\hat{\theta }_{\text{RB}}}$ are linear in *X*_(1)_ and *X*_(*n*)_, but with different coefficients. This will be shown in two steps: first, two unbiased estimators $\hat{\theta }_m$ and $\hat{\theta }_M$ of θ will be defined, as constant multiples of (the minimum) *X*_(1)_ and (the maximum) *X*_(*n*)_, respectively. Second, among the affine combinations of $\hat{\theta }_m$ and $\hat{\theta }_M$, a minimum-variance solution ${\hat{\theta }_{\text{LV}}}$ will be found, with strictly lower variance than ${\hat{\theta }_{\text{RB}}}$, uniformly in θ > 0.

This idea of taking an affine combination of two unbiased estimators to define a new unbiased estimator appeared at least 200 years ago in a work by Laplace (see Stigler 1973), and is still found in modern use (e.g., see recent work by Damilano and Puig 2004).

As a preliminary, let *Y*_1_, *Y*_2_, …, *Y_n_* be iid, with common *U*(*a*, *b*) distribution, for some *a* < *b*, with *Y*_(1)_ = min (*Y_i_*) and *Y*_(*n*)_ = max (*Y_i_*). Then

(2) E[Y(1)]=a+1n+1(b-a)E[Y(n)]=b-1n+1(b-a)\begin{eqnarray*} E[Y_{(1)}] & = & a+{1 \over {n+1}}(b-a) \ \nonumber\\ E[Y_{(n)}]&=&b-{1 \over {n+1}}(b-a) \\ \end{eqnarray*}

(3) var[Y(1)]=var[Y(n)]=n(n+1)2(n+2)(b-a)2\begin{eqnarray*} \text{var}[Y_{(1)}] & = & \text{var}[Y_{(n)}] = {n \over {(n+1)^2(n+2)}}(b-a)^2 \\ \end{eqnarray*}

(4) cov[Y(1),Y(n)]=1(n+1)2(n+2)(b-a)2ρ[Y(1),Y(n)]=1n.\begin{eqnarray*} \text{cov}[Y_{(1)}, Y_{(n)}] & = & {1 \over {(n+1)^2(n+2)}}(b-a)^2 \ \nonumber\\ \rho [Y_{(1)}, Y_{(n)}]&=&{1 \over n}. \end{eqnarray*}

Step 1: Substituting *a* = (1 − *k*)θ and *b* = (1 + *k*)θ, the last formulas take the form

(5) Eθ[X(1)]=1-n-1n+1kθEθ[X(n)]=1+n-1n+1kθ\begin{eqnarray*} E_{\theta }[ {{X_{(1)}}} ] & = & \left({1 - {{n - 1} \over {n + 1}}k} \right)\theta \ \nonumber\\ E_{\theta }[ {{X_{(n)}}} ] &=& \left({1 + {{n - 1} \over {n + 1}}k} \right)\theta \\ \end{eqnarray*}

(6) varθ[X(1)]=varθ[X(n)]=4nk2θ2(n+1)2(n+2)\begin{eqnarray*} \text{var}_{\theta }[X_{(1)}] & = & \text{var}_{\theta }[X_{(n)}]= {{4 n k^2 \theta ^2} \over {(n+1)^2 (n+2)}} \\ \end{eqnarray*}

(7) covθ[X(1),X(n)]=4k2θ2(n+1)2(n+2)ρθ[X(1),X(n)]=1n\begin{eqnarray*} \text{cov}_{\theta }[X_{(1)}, X_{(n)}] & = & {{4 k^2 \theta ^2} \over {(n+1)^2 (n+2)}} \ \nonumber\\ \rho _{\theta }[X_{(1)}, X_{(n)}]&=&{1 \over n} \end{eqnarray*}

giving rise to the “basic” unbiased estimators of θ

(8) θ^m=11-n-1n+1kX(1)θ^M=11+n-1n+1kX(n)\begin{eqnarray*} \hat{\theta }_m & = & \frac{1}{{1 - \frac{{n - 1}}{{n + 1}}k}} X_{(1)} \nonumber \\ \hat{\theta }_M & = & \frac{1}{{1 + \frac{{n - 1}}{{n + 1}}k}} X_{(n)} \end{eqnarray*}

with variances

(9) varθ[θ^m]=4nk2θ2((1-k)(n-1)+2)2(n+2)varθ[θ^M]=4nk2θ2((1+k)(n-1)+2)2(n+2).\begin{eqnarray*} \text{var}_\theta [\hat{\theta }_m] & = & {{4 n k^2 \theta ^2} \over {((1\bm {-} k) (n-1) + 2)^2}(n+2)} \nonumber \\ \text{var}_\theta [\hat{\theta }_M] & = & {{4 n k^2 \theta ^2} \over {((1 \bm {+} k) (n-1) + 2)^2}(n+2)}. \end{eqnarray*}

These variances reveal that *X*_(*n*)_ is strictly more informative about θ than *X*_(1)_ throughout the range of *n* and *k*. Indeed, the variance of $\hat{\theta }_M$ is uniformly smaller than the variance of $\hat{\theta }_m$, even asymptotically:

(10) limn→∞n2varθ[θ^m]=4k2(1-k)2θ2>4k2(1+k)2θ2=limn→∞n2varθ[θ^M].\begin{eqnarray*} \lim _{n \rightarrow \infty } n^2 \text{var}_\theta [\hat{\theta }_m] &=& {{4 k^2} \over {(1\bm {-}k)^2}}{\theta ^2} > {{4 k^2} \over {(1\bm {+}k)^2}}{\theta ^2} \nonumber\\ &=&\lim _{n \rightarrow \infty } n^2 \text{var}_\theta [\hat{\theta }_M]. \end{eqnarray*}

Step 2: When *k* < 1, the basic estimators $\hat{\theta }_m$ and $\hat{\theta }_M$ generate the more general family of unbiased estimators $\hat{\theta }_{mM}^{(\alpha)}$ of θ obtained as their affine combinations

(11) θ^mM(α)=(1-α)θ^m+αθ^M=1-α1-n-1n+1kX(1)+α1+n-1n+1kX(n)\begin{eqnarray*} \hat{\theta }_{mM}^{(\alpha)} &=& (1 - \alpha) \hat{\theta }_m + \alpha \hat{\theta }_M \nonumber\\ &=& \frac{1-\alpha }{{1 - \frac{{n - 1}}{{n + 1}}k}} X_{(1)} + \frac{\alpha }{{1 + \frac{{n - 1}}{{n + 1}}k}} X_{(n)} \end{eqnarray*}

from which it is easy to identify the Rao–Blackwell improvement ${\hat{\theta }_{\text{RB}}}$ of *X*_1_ as the case with $\alpha =\alpha _{\text{RB}}={1 \over 2}({1 + \frac{{n - 1}}{{n + 1}}k}) > {1 \over 2}$, and ${\hat{\theta }_{\text{LV}}}$ as the standard analytically derived case with

(12) α=αLV=var[θ^m]-cov[θ^m,θ^M]var[θ^m]+var[θ^M]-2cov[θ^m,θ^M]\begin{eqnarray*} \alpha =\alpha _{\text{LV}}={{\text{var}[\hat{\theta }_m]-\text{cov}[\hat{\theta }_m,\hat{\theta }_M]} \over {\text{var}[\hat{\theta }_m]+\text{var}[\hat{\theta }_M]- 2 \text{cov}[\hat{\theta }_m,\hat{\theta }_M]}} \end{eqnarray*}

and minimal variance

(13) var[θ^mM(αLV)]=var[(1-αLV)θ^m+αLVθ^M]=var[θ^m]var[θ^M]-cov2[θ^m,θ^M]var[θ^m]+var[θ^M]-2cov[θ^m,θ^M]\begin{eqnarray*} \text{var}[\hat{\theta }_{mM}^{(\alpha _{\text{LV}})}]&=&\text{var}[(1-\alpha _{\text{LV}}) \hat{\theta }_m + \alpha _{\text{LV}} \hat{\theta }_M]\nonumber\\[4pt] &=&{{\text{var}[\hat{\theta }_m] \text{var}[\hat{\theta }_M]-\text{cov}^2[\hat{\theta }_m,\hat{\theta }_M]} \over {\text{var}[\hat{\theta }_m]+\text{var}[\hat{\theta }_M]- 2 \text{cov}[\hat{\theta }_m,\hat{\theta }_M]}} \end{eqnarray*}

that in the present case becomes

(14) αLV=12(1+2n(n-1)k+(n+1)1k),\begin{eqnarray*} \alpha _{\text{LV}} = \frac{1}{2} (1 + {{2 n} \over {(n-1) k + (n+1){1 \over k}}}), \end{eqnarray*}

which yields the following two representations for ${{\hat{\theta }}_{\text{LV}}}$

(15) θ^LV=12-n(n-1)k+(n+1)1kθ^m+12+n(n-1)k+(n+1)1kθ^M=12(k2(n-1)(n+1)+1)[(1-k)X(1)+(1+k)X(n)]\begin{eqnarray*} {{\hat{\theta }}_{\text{LV}}} & = & \left({\frac{1}{2} - \frac{n}{{(n - 1)k + (n + 1)\frac{1}{k}}}} \right){{\hat{\theta }}_m} \nonumber\\ &&+ \left({\frac{1}{2} + \frac{n}{{(n - 1)k + (n + 1)\frac{1}{k}}}} \right){{\hat{\theta }}_M} \nonumber \\ & = & \frac{1}{{2({{k^2}\frac{{({n - 1})}}{{({n + 1})}} + 1})}}[ {({1 - k}){X_{(1)}} + ({1 + k}){X_{(n)}}} ] \end{eqnarray*}

with

(16) var[θ^LV]=2k2θ2((1+k2)(n-1)+2)(n+2).\begin{eqnarray*} \text{var}[\hat{\theta }_{\text{LV}}]={{2 k^2 \theta ^2} \over {((1+k^2)(n-1) +2)(n+2)}}. \end{eqnarray*}

The asymptotic standard deviation $\sigma [\hat{\theta }_{\text{LV}}]$, proportional to $k \sqrt{{2 \over {1+k^2}}}$, is smaller than $\sigma [\hat{\theta }_M]$, proportional to ${{2 k} \over {1+k}}$. However, their asymptotic ratio ranges from only $\sqrt{2}$ (as *k*↓0) to 1 (as *k*↑1), which means that the two estimators $\hat{\theta }_M$ and $\hat{\theta }_{\text{LV}}$ are of comparable accuracy throughout the range, while $\hat{\theta }_m$ becomes infinitely worse as *k* → 1.

We proceed now to analyze the Rao–Blackwell improvement $\hat{\theta }_{\text{RB}}$. From a probability point of view (i.e., when θ is a known constant), *E*_θ_[*X*_1_|*X*_(1)_, *X*_(*n*)_] treats *X*_(1)_ and *X*_(*n*)_ symmetrically. Indeed, the two are homoscedastic and stochastically equidistant from θ. From a statistics point of view (i.e., when *learning about* θ), *X*_(*n*)_ is more informative than *X*_(1)_, in the sense that θ is unbiasedly estimated more accurately by contracting *X*_(*n*)_ than by expanding *X*_(1)_. Thus, $\hat{\theta }_M$ should be intuitively expected to have a more decisive weight.

To see that indeed $\alpha _{\text{RB}}<\alpha _{\text{LV}}$, it is needed to check that ${{n-1} \over {n+1}} k < {{2 n} \over {(n-1) k + (n+1){1 \over k}}}$ or equivalently that $1 < {{2 n} \over {(n-1)(1 + k^2 {{n-1} \over {n+1}})}}$. But this last expression is monotone decreasing in *k*, with value ${{n+1} \over {n-1}}>1$ at *k* = 1.

The variance of $\hat{\theta }_{\text{RB}}$ can be expressed as

(17) var [θ^RB]=2k2θ2(n+2)(n+1).\begin{eqnarray*} {\mathop {\rm var}} [{{{\hat{\theta }}_{\text{RB}}}}] = \frac{{2{k^2}}{\theta ^2}}{{({n + 2}){{({n + 1})}}}}. \end{eqnarray*}

The ratio $ { {\mathop {\rm var}} [{{{\hat{\theta }}_{\text{RB}}}}] \over {\mathop {\rm var}} [{{{\hat{\theta }}_{\text{LV}}}}] } = 1+k^2 {(n-1) \over (n+1)}$ increases with *n* and, like ${ {\mathop {\rm var}} [{{{\hat{\theta }}_{M}}}] \over {\mathop {\rm var}} [{{{\hat{\theta }}_{\text{LV}}}}] }$, is bounded from above by 2.

### 2.1 A Remark on Minimal But NonComplete Sufficient Statistics

The scale-uniform example given illustrates that using the Rao–Blackwell theorem with a noncomplete minimal sufficient statistic on a crude initial (unbiased) estimator does not always yield an estimator with the lowest possible variance. And in fact, it never will. Any unbiased estimator that is a function of the minimal sufficient statistic is its own Rao–Blackwell improvement. If one such estimator has larger variance than another (such as $\hat{\theta }_m$ vs. $\hat{\theta }_M$), then the first has been improved, and if the two have equal variances, their average improves both. This is in essence the message in Torgersen’s converse to the Rao–Blackwell theorem (Torgersen 1988).

## 3.INEFFICIENCY OF THE MAXIMUM LIKELIHOOD ESTIMATOR

Under the usual differentiability assumptions, MLEs converge to the true parameter value at rate $n^{-{1 \over 2}}$ *efficiently*, that is, with asymptotic variance that achieves the Crámer–Rao lower bound for the variance of unbiased estimators. In the model at hand these assumptions are violated and the parameter θ can be estimated, as seen above, with a faster rate of consistency *n*^− 1^.

For the scale-uniform family, the likelihood function $({1 \over {2 k \theta }})^n I_{\lbrace {{X_{(n)} \over {1+k}}} < \theta < {X_{(1)} \over {1-k}}\rbrace }$ is maximized at the minimal feasible θ value $\hat{\theta }_{\text{MLE}} = {1 \over {1+k}} X_{(n)}$, a slight departure from its unbiased correction $\hat{\theta }_M = \frac{1}{{1 + \frac{{n - 1}}{{n + 1}}k}} X_{(n)}$ developed above. Thus, the asymptotic variance of $n (\hat{\theta }_{\text{MLE}} - \theta)$ is ${{4 k^2} \over {(1+k)^2}}{\theta ^2}$, strictly bigger than the asymptotic variance ${{2 k^2} \over {1+k^2}}{\theta ^2}$ achieved by $n (\hat{\theta }_{\text{LV}} - \theta)$, showing that MLE is inefficient in this case. The likelihood function discloses valuable deterministic information about θ, its feasible Low endpoint $L={{X_{(n)} \over {1+k}}}$ and High endpoint $H={{X_{(1)} \over {1-k}}}$. The MLE, $\hat{\theta }_{\text{MLE}} = L$, ignores the information provided by *X*_(1)_ and pays a price for it in terms of efficiency.

The likelihood function exhibits a nice feature of the Rao–Blackwell improvement $\hat{\theta }_{\text{RB}} = {{1+k} \over 2} L + {{1-k} \over 2} H$: it is the *only* unbiased estimator of θ in the class $\hat{\theta }_{mM}^{(\alpha)}$ that is guaranteed to obtain values in the feasibility interval [*L*, *H*], since it is the only one with coefficients adding up to 1 when viewed as a linear combination of *L* and *H*. Of course, feasibility is guaranteed for MLE (= *L*) and Bayesian estimators, the subject of the next section.

## 4.AN UNBIASED IMPROPER BAYES ESTIMATOR

In this section, we intend to complement the previous section by showing that, while MLE leads to the inefficient $\hat{\theta }_M$, Bayes estimation leads to a slight improvement over $\hat{\theta }_{\text{LV}}$, so the latter is a reasonably good estimator and the requirement of unbiasedness does not take a heavy toll. However, as will be seen, $\hat{\theta }_{\text{LV}}$ is uniformly dominated by another unbiased estimator, one that is not a linear combination of *X*_(1)_ and *X*_(*n*)_.

**Figure 1:**
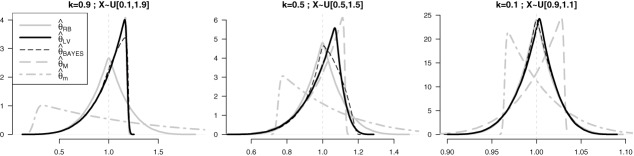
The simulated distribution (10^6^ runs, sample size *n* = 5) of various unbiased estimators for θ = 1 with design parameter *k* = 0.9; 0.5; 0.1.

Letting ϑ stand for the Bayes-oriented random scale parameter and θ stand for its possible values, assume a smooth (improper) prior density λ(θ) on ϑ, proportional to θ^− *a*^. As far as the posterior density of ϑ is concerned, it is only needed for its support, the feasibility interval $[L, H]=[{{X_{(n)} \over {1+k}}}, {X_{(1)} \over {1-k}}]$, where it becomes a proper posterior density from the first observation onward. The Bayes estimator of the scale parameter θ, the posterior expectation of ϑ, is

(18) θ^BAYES(a)=∫LHθλ(θ)(2kθ)ndθ∫LHλ(θ)(2kθ)ndθ=∫LHdθθn-1+a∫LHdθθn+a=n-1+an-2+aL-(n-2+a)-H-(n-2+a)L-(n-1+a)-H-(n-1+a)=n-1+an-2+a1-HL-1(HL)n-1+a-1L.\begin{eqnarray*} \hat{\theta }_{\text{BAYES}}^{(a)} & = & {{\int _L^H {{\theta \lambda (\theta)} \over {(2 k \theta)^n}} d\theta } \over {\int _L^H {{\lambda (\theta)} \over {(2 k \theta)^n}} d\theta }} \nonumber\\ &=& {{\int _L^H {d\theta \over {\theta }^{n-1+a}}} \over {\int _L^H {{d\theta } \over {{\theta }^{n+a}}}}} = {{n-1+a} \over {n-2+a}} {{L^{-(n-2+a)}-H^{-(n-2+a)}} \over {{L^{-(n-1+a)}-H^{-(n-1+a)}}}} \nonumber \\ & = & {{n-1+a} \over {n-2+a}} \left[1 - {{{H \over L}-1} \over {({H \over L})^{n-1+a} -1}}\right] L. \end{eqnarray*}

The Bayes estimators under this conjugate bounded Pareto family are homogenous of order 1, that is, share with *X*_1_, $\overline{X}_n$, $\hat{\theta }_{\text{RB}}$, and $\hat{\theta }_{\text{LV}}$ their “respect” for the scale-parameter nature of θ, the property that the distribution of ${{\hat{\theta }} \over {\theta }}$ is a pivotal quantity, that may depend on *n* and *k* but is independent of θ. Accordingly, their bias and standard deviation are constant multiples of θ. We show in the sequel that for the case *a* = 2, the Bayes estimator (henceforth $\hat{\theta }_{\text{BAYES}}^{(2)}=\hat{\theta }_{\text{BAYES}}$ for short) is unbiased, simultaneously on *n* ⩾ 2 and *k* ∈ (0, 1).

The joint density of *X*_(1)_ and *X*_(*n*)_ is $f_{\theta }(x,y)={{n(n-1)} \over {(2 k \theta)^n}}(y-x)^{n-2}$ on [(1 − *k*)θ, (1 + *k*)θ]. The expectation of $\hat{\theta }_{\text{BAYES}}$ can be evaluated for θ = 1 as follows:

(19) E[θ^BAYES]=n+1nEX(n)1+k-n(n-1)(2k)n×∫1-k1+k∫x1+ky1+k1+k1-kxy-1(1+k1-kxy)n+1-1×X(n)1+k(y-x)n-2dydx.\begin{eqnarray*} E[\hat{\theta }_{\text{BAYES}}] &=& {{n+1} \over n} \left[E \left[{{X_{(n)}} \over {1+k}}- {{n(n-1)} \over {(2 k)^n}}\right. \right. \nonumber\\ &&\times \int _{1-k}^{1+k} \int _x^{1+k} {{y} \over {1+k}} {{{ {{1+k} \over {1-k}}}{x \over y} -1} \over {({{1+k} \over {1-k}}{x \over y})^{n+1} -1}}\nonumber\\ && \left. \times {\vphantom{{{X_{(n)}} \over {1+k}}}} (y-x)^{n-2} dy dx\right]. \end{eqnarray*}

The proof that $E[\hat{\theta }_{\text{BAYES}}] \equiv 1$ appears in the Appendix.

Figure 1 displays the empirical distribution of the various unbiased estimators of θ for sample size *n* = 5 from the uniform-scale distribution with θ = 1 and *k* = 0.9; 0.5; 0.1.

The three panels display that $\hat{\theta }_{\text{RB}}$ (solid gray line) is symmetrically distributed around 1, with $\hat{\theta }_{m}$ (two-dash gray line) skewed to the right and $\hat{\theta }_{M}$ (long-dash gray line) skewed to the left. Both $\hat{\theta }_{\text{LV}}$ (solid line) and $\hat{\theta }_{\text{BAYES}}$ (thin dashed line) overlap $\hat{\theta }_{M}$ as *k*↑1 (leftmost panel), and approach $\hat{\theta }_{\text{RB}}$ as *k*↓0 (rightmost panel).

**Figure 2:**
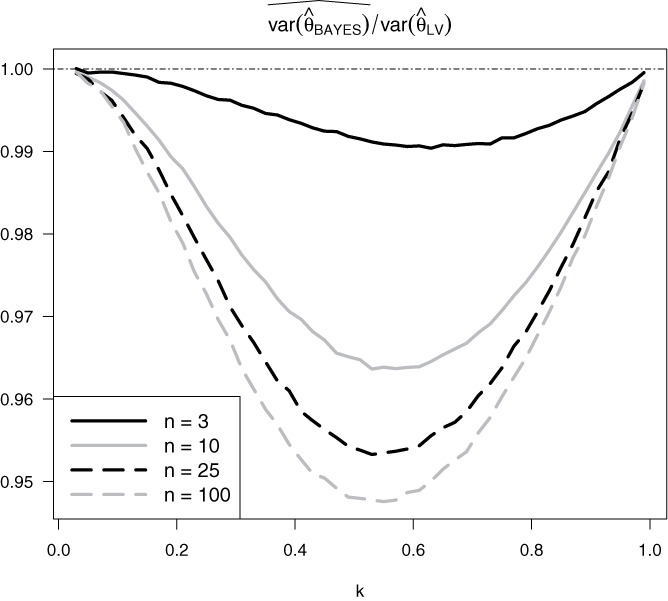
The ratio of the empirical variance of $\hat{\theta }_{\text{BAYES}}$ (on 10^8^ runs each) to the theoretical variance of $\hat{\theta }_{\text{LV}}$ for various sample sizes over a range of *k* values.

Since generalized Bayes rules need not be admissible, we cannot state from general principles that $\hat{\theta }_{\text{BAYES}}$ has lower variance than $\hat{\theta }_{\text{LV}}$. However, Figure 2 provides evidence that it does. Figure 2 displays for θ = 1, all *k* that are multiples of ${1 \over 50}$ and the choices *n* = 3, 10, 25, 100 of the sample size, the ratio of the empirical variance of $\hat{\theta }_{\text{BAYES}}$ (on 10^8^ runs each, using the R software (R Core Team 2014)) to the theoretical variance of $\hat{\theta }_{\text{LV}}$ (16). These graphs are U-shaped, with value 1 toward the endpoints. The larger *n* is, the stronger the improvement of $\hat{\theta }_{\text{BAYES}}$ over $\hat{\theta }_{\text{LV}}$, but seemingly never by as much as 10%.

## 5.DISCUSSION

The concepts of minimal sufficiency and completeness of parametric families, at the basis of the theory of statistics, allow for the development of “optimal” statistical methods under proper sufficient conditions. The above example serves to point out the difficulty of exhibiting a “uniformly best estimator” in some settings. The purpose in presenting it is primarily pedagogical.

This article introduced the *scale-uniform* family of distributions *U*((1 − *k*)θ, (1 + *k*)θ) (with unknown mean θ > 0 and a known design parameter *k* ∈ (0, 1)). This family helps to illustrate the limitations of the Rao–Blackwell improvement when using a sufficient statistic that is minimal but not complete. It also serves to show that the maximum likelihood estimator may be inefficient for finite samples as well as asymptotically. Cases with inefficient MLE for finite samples are easily available: when estimating λ for the exponential distribution, the unbiased estimator ($\frac{n-1}{n} \frac{1}{\bar{X}}$) has lower variance than the MLE ($\frac{1}{\bar{X}}$). However, an *asymptotically* inefficient MLE is harder to come by.

The impressive progress in statistics in the last few decades has dimmed the role played by minimal sufficiency and completeness of parametric families in the development of statistical tools. Various nonparametric methods ranging from bootstrap and permutation tests to random forests and deep learning have emerged, playing now prominent roles in statistical practice. Nevertheless, the need for parametric methods continues to surface in this new era. For example, new online machine learning methods are used in cases where the data become available in a sequential fashion. Since these methods need to be scalable for petabytes of information, data compression is essential (such as used by Google, Facebook, etc.). Another example is the use of map-reduce techniques that rely on summary statistics, either for efficiency in distributed computing, or for privacy-preserving properties (e.g., in hospital data, as is currently done in the medical informatics platform of the European Human Brain Project). A last example is of modern health care where the hope is to discover personalized medicines that can best treat the conditions of a specific individual. Such specificity is usually determined through inference, based on finding and analyzing a relatively small homogenous subsample of patients. In all three examples, the methods used often rely on minimal sufficient statistics, and whether this statistic is complete or not has consequences on the “optimal” properties of such methods. Hence, we would argue that while traditional statistical methods may have less of a need for old-school parametric assumptions, this need continues to resurface as we face new challenges in the modern era of massive, online, distributed, private, and personalized data.

## APPENDIX: PROOFS

### A.1. Proof of ${\rho }\left[ {{Y_{\left(1 \right)}},{Y_{\left(n \right)}}} \right] = \frac{1}{n}$

For *n* iid *Y_i_* ∼ *U*(*a*, *b*), the *n* − 1 observations except for *Y*_(1)_ are conditionally iid given *Y*_(1)_, distributed *U*(*Y*_(1)_, *b*). Therefore $E\left[ {{Y_{\left(n \right)}}|{Y_{\left(1 \right)}}} \right] = \frac{{ {n - 1}}}{n} b + \frac{1}{n}{Y_{\left(1 \right)}}$. Since this conditional expectation is linear, its slope ${1 \over n}$ coincides with the slope of the linear regression that, for dependent and independent variables with equal variances is equal to their correlation coefficient.

### A.2. Proof that $\hat{\theta }_{\text{BAYES}}$ is Unbiased

Apply $E[{{X_{(n)}} \over {1+k}}]=1-{{2 k} \over {(1+k)(n+1)}}$, transform variables

(20) u=y-x;v=xyx=uv1-v;y=u1-v\begin{eqnarray*} u & = & y-x \ ; \ v = {x \over y} \nonumber \\ x & = & {{u v} \over {1- v}} \ ; \ y={u \over {1- v}} \end{eqnarray*}

and evaluate the Jacobian as $|\det {{\partial (x,y)} \over {\partial (u,v)}}|={u \over {(1-v)^2}}$. The integrand of the double integral in (19) is transformed into a function in which the new variable *u* appears only multiplicatively as *u^n^*, and the inner integration over *u* can be easily performed once the endpoints of *u* are identified, for fixed *v*. For this purpose, *u* is represented in terms of *v* and *x* as $u={{1-v} \over v} x$. Then its lower endpoint $u_* = (1-k) {{1-v} \over v}$ is obtained by the substitution *x* = 1 − *k* while its upper endpoint *u** = (1 + *k*)(1 − *v*) is obtained from the equation resulting from the substitution *x* = 1 + *k* − *u*. The inner integral is

(21) ∫u*u*undu=1n+1((u*)n+1-(u*)n+1)=1n+11-vvn+1(1+k)n+1vn+1-(1-k)n+1.\begin{eqnarray*} \int _{u_*}^{u^*} u^n du &=& {1 \over {n+1}}((u^*)^{n+1}-(u_*)^{n+1}) \nonumber\\ &=& {1 \over {n+1}}\left({{1-v} \over v}\right)^{n+1}\left((1+k)^{n+1} v^{n+1} \right.\nonumber\\ &&-\left. (1-k)^{n+1}\right). \end{eqnarray*}

Putting these elements together, (19) becomes

(22) E[θ^BAYES]=1+1-k1+k1n-(n-1)(1-k)n+1(2k)n(1+k)×∫1-k1+k11+k1-kv-1(1-v)n-2vn+1dv,\begin{eqnarray*} E[\hat{\theta }_{\text{BAYES}}]&=&1+{{1-k} \over {1+k}}{1 \over n} - {{(n-1)(1-k)^{n+1}} \over {(2 k)^n (1+k)}} \nonumber\\ &&\times \int _{{1-k} \over {1+k}}^1 {{\left({{1+k} \over {1-k}} v-1\right) (1-v)^{n-2}} \over {v^{n+1}}} dv, \end{eqnarray*}

which upon the rescaling $w={{(1+k) v -(1-k)} \over {2 k}}$ becomes

(23) E[θ^BAYES]=1+1-k1+k1n-(n-1)(1-k)n(1+k)(2k)n+1×∫01w(1-w)n-2(w+1-k2k)n+1dw.\begin{eqnarray*} E[\hat{\theta }_{\text{BAYES}}]&=&1+{{1-k} \over {1+k}}{1 \over n} - {{(n-1)(1-k)^n (1+k)} \over {(2 k)^{n+1}}} \nonumber\\ &&\times \int _0^1 {{w (1-w)^{n-2}} \over {(w+{{1-k} \over {2 k}})^{n+1}}} dw. \end{eqnarray*}

For $E[{\hat{\theta }_{\text{BAYES}}}] = 1$ to hold (meaning that $\hat{\theta } _{\text{BAYES}}$ is unbiased), the identity

(24) ∫01w(1-w)n-2(w+1-k2k)n+1dw=(2k)n+1n(n-1)(1-k)n-1(1+k)2\begin{eqnarray*} \int _0^1 {\frac{{w{{(1 - w)}^{n - 2}}}}{{{{(w + \frac{{1 - k}}{{2k}})}^{n + 1}}}}} dw = \frac{{{{(2k)}^{n + 1}}}}{{n(n - 1){{(1 - k)}^{n - 1}}{{(1 + k)}^2}}} \nonumber \\[-4pt] \end{eqnarray*}

must hold. This formula appears explicitly in Gradshteyn & Ryzhik (Jeffrey and Zwillinger 2007, formula 3.197 (4)) as

(25) ∫01xμ-1(1-x)ν-1(1+ax)-μ-νdx=(1+a)-μB(μ,ν),\begin{eqnarray*} &&\int _0^1 x^{\mu -1}(1-x)^{\nu -1}(1+ax)^{-\mu -\nu }dx\nonumber\\ &&\quad =(1+a)^{-\mu } B(\mu,\nu), \end{eqnarray*}

where *B* is the Beta function *B*(μ, ν) = ∫^1^_0_*t*^μ − 1^(1 − *t*)^ν − 1^ *dt*. Substituting *x* = *w*, μ = 2, ν = *n* − 1, $a= \frac{2k}{1-k}$ (after its inverse is taken out of the integral), identity (A.5) follows.

The above formula can also be accessed through *WolframAlpha.com* (LLC 2014).
